# Transcranial Magnetic Stimulation Enhances the Therapeutic Effect of IGF-Trap in Intracerebral Glioma Models

**DOI:** 10.3390/ph17121607

**Published:** 2024-11-28

**Authors:** Stephanie Perrino, Udi Vazana, Ofer Prager, Lior Schori, Gal Ben-Arie, Anna Minarik, Yinhsuan Michely Chen, Orçun Haçariz, Masakazu Hashimoto, Yiftach Roth, Gabriel S. Pell, Alon Friedman, Pnina Brodt

**Affiliations:** 1 The Research Institute, The McGill University Health Center, Montreal, QC H4A 3J1, Canada; stephanie.perrino@affiliate.mcgill.ca (S.P.);; 2 Departments of Physiology and Cell Biology, Cognitive and Brain Sciences, The Zelman Center for Brain Science Research, Ben-Gurion University of the Negev, 1 Ben-Gurion Blvd., Beer-Sheva 8410501, Israel; udi.vazana@gmail.com (U.V.); pragero@post.bgu.ac.il (O.P.); alon.friedman@dal.ca (A.F.); 3 Faculty of Health Sciences, Ben-Gurion University of the Negev, 1 Ben-Gurion Blvd., Beer-Sheva 8410501, Israel; 4 Department of Radiology, Soroka Medical Center, Beer-Sheva 8410501, Israel; 5 Department of Medical Neuroscience and the Brain Repair Centre, Faculty of Medicine, Dalhousie University, 5850 College St., Halifax, NS B3H 4R2, Canada; 6 Department of Medicine, Division of Experimental Medicine, McGill University, Montreal, QC H3A 0G4, Canada; 7 Department of Surgery, Division of Experimental Surgery, McGill University, Montreal, QC H3A 0G4, Canada; 8 Brainsway Ltd., 19 Hartom St., Jerusalem 9777518, Israel; yiftach@brainsway.com (Y.R.); gabi@brainsway.com (G.S.P.)

**Keywords:** glioma, animal models, rTMS, IGF, IGF-Trap, therapy

## Abstract

**Background:** Glioblastoma multiforme is an aggressive malignancy with a dismal 5-year survival rate of 5–10%. Current therapeutic options are limited, due in part to drug exclusion by the blood–brain barrier (BBB). We have previously shown that high-amplitude repetitive transcranial magnetic stimulation (rTMS) in rats allowed the delivery across the BBB of an IGF signaling inhibitor—IGF-Trap. The objective of this study was to assess the therapeutic effect of IGF-Trap when delivered in conjunction with rTMS on the intracerebral growth of glioma. **Results**: We found that systemic administration of IGF-Trap without rTMS had a minimal effect on the growth of orthotopically injected glioma cells in rats and mice, compared to control animals injected with vehicle only or treated with sham rTMS. In rats treated with a combination of rTMS and IGF-Trap, we observed a growth retardation of C6 tumors for up to 14 days post-tumor cell injection, although tumors eventually progressed. In mice, tumors were detectable in all control groups by 14–17 days post-injection of glioma GL261 cells and progressed rapidly thereafter. In mice treated with rTMS prior to IGF-Trap administration, tumor growth was inhibited or delayed, although the tumors also eventually progressed. **Conclusion**: The results showed that rTMS could increase the anti-tumor effect of IGF-Trap during the early phases of tumor growth. Further optimization of the rTMS protocol is required to improve survival outcomes.

## 1. Introduction

Glioblastoma multiforme (GBM) is the most prevalent primary brain malignancy. Its incidence increases in adults 55 years and older, and it is associated with a dismal 5-year survival of 5–10% [1]. GBM is characteristically poorly differentiated and highly heterogeneous [2]. Among the molecular/genomic alterations identified in this disease are amplifications in tyrosine kinase receptors such as the epidermal growth factor receptor (EGFR), platelet-derived growth factor receptor (PDGFR) and type 1 insulin-like growth factor receptor (IGF-1R) [2]. In 86–89.6% of GBMs, the PI3K/Akt pathway is altered, identifying it, and its upstream receptor tyrosine kinases, including IGF-1R, as potential therapeutic targets [3]. Epigenetic changes in the retinoblastoma (Rb) gene and mutations and copy number alterations in the tumor suppressor p53 have also been identified [4]. Overexpression of IGF-1R has been linked to poorer survival in GBM patients [5,6] and IGF signaling was implicated in GBM resistance to chemotherapy [6] and to treatment with EGFR [7] and PDGFR [8] inhibitors.

The IGF-1R ligands, which are insulin-like growth factors (IGF-1 and IGF-2), are involved in cell growth and differentiation. Ligand binding triggers IGF-1R signaling via the PI3K/Akt and MEK/ERK pathways, as well as nuclear IGF-1R transport to activate gene transcription, thereby regulating resistance to apoptosis, cell proliferation, and cell cycle progression, as well as promoting tumor progression [6,9,10]. We and others have shown that targeting IGF-1R inhibits the growth of pediatric and adult GBM cells in vitro and in vivo [6,11,12]. Recently, we reported that the intracerebral growth of adult GBM cells with genetically engineered downregulation of IGF-1R was inhibited [13]. However, to date, IGF-1R targeting in GBM has not advanced to clinical practice.

We previously reported on the bioengineering of a potent IGF-1R signaling inhibitor, the IGF-Trap. The IGF-Trap is a fusion protein consisting of the entire extracellular domain of human IGF-1R fused to the Fc domain of human IgG_1_. The IGF-Trap binds to IGF-1 and IGF-2 (but not insulin) with high affinity, thereby reducing ligand bioavailability to the cognate receptor and impeding receptor activation [14,15]. We have shown that IGF-Trap could inhibit the growth of several very aggressive carcinomas, including colon, lung and pancreatic carcinoma cells, in vivo [14,15]. A highly effective variant of the IGF-Trap, with increased stability and efficacy, was subsequently engineered [16] (reviewed in [14]). The effect of this inhibitor on GBM growth remains to be assessed.

One major challenge to successful treatment of GBM and other brain malignancies is effective drug delivery to the brain, as drug exclusion by the blood–brain barrier (BBB) limits drug access to the tumor site. The BBB is a semi-permeable layer of endothelial cells that protects the brain from foreign and potentially harmful substances. Hence, there has been a concerted effort by scientists and clinicians to develop strategies aimed at transiently increasing the permeability of the BBB in a safe manner, with minimal adverse effects for drug delivery [17,18]. To date, strategies BBB disruption include intra-arterial infusion of mannitol at hyperosmotic concentrations, which is associated with significant morbidity [19,20], and focused ultrasound, which has advanced to clinical trials [21,22,23,24,25]. In the latter approach, ultrasound is administered to the brain in combination with intravenous injection of microbubbles, which disrupts the endothelium’s tight junctions. However, the resultant BBB disruption may be associated with injury to brain microvessels, microbleeds [26] and brain inflammation [27]. Thus, the field of controlled BBB opening for enhanced drug delivery is still experimental, and no method has shown both the efficacy and safety levels required for routine clinical use.

Transcranial magnetic stimulation (TMS), a non-invasive brain stimulation approach, generates short (typically 200–400 µs) magnetic field pulses, inducing an electric field that depolarizes large groups of neurons when delivered at appropriate magnitude and orientation. Deep TMS, a recently developed technology enabling the stimulation of deeper brain regions than standard TMS [28], is already an FDA-approved approach for the treatment of depression [29], as well as comorbid anxiety [30], obsessive compulsive disorder [31] and smoking cessation [32]. Treatment of neuropsychiatric conditions with TMS involves multiple applications of repeated pulses at *controlled* intensities. This approach, known as repetitive TMS (rTMS), is believed to influence the brain via mechanisms related to synaptic plasticity. In contrast to these applications, we have previously shown that a single application of rTMS to the brain in rats, at *high* intensity, induced a transient BBB opening and facilitated drug delivery to the cortex [33]. We used neuroimaging to confirm an increase in BBB permeability using deep TMS in a pilot clinical study performed in patients with malignant glial tumors [33]. In follow-up studies, we have recently shown that the technique induced a safe and transient disruption of the BBB and could facilitate the delivery across the BBB of a large-sized biologic, such as the IGF-Trap, in healthy rats [34]. Here, we used rat and mouse orthotopic tumor models to further evaluate the safety of brain rTMS and determine whether an rTMS-induced increase in IGF-Trap delivery to the brain could inhibit the growth of intra-cerebrally implanted brain tumors. We show that rTMS, at the frequency used, was safe in both rats and mice. Furthermore, we show that when systemic IGF-Trap administration was combined with rTMS, the growth of glioma cells implanted in the brain was inhibited in both rats and mice, although the effect was transient. Further optimization of the treatment protocol may be required to enhance the therapeutic effect and improve survival.

## 2. Results

### 2.1. Repeated Low-Frequency rTMS Does Not Induce Brain Injury or Neurobehavioral Impairment

**Figure 1:**
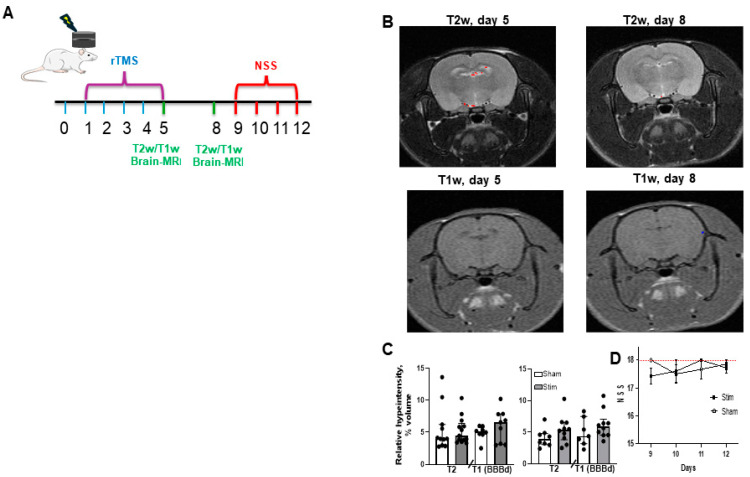
Repeated low-frequency rTMS is safe. (**A**) shows a diagrammatic depiction of the experimental protocol. ((**B**)-**top**) shows T2-weighted (T2w) coronal rat brain MRI acquired after five consecutive days of repeated low-frequency rTMS (day 5, **left**) and three days later (day 8, **right**), overlaid with detection of hyper-intensified voxels; and ((**B**)-**bottom**) shows T1-weighted (T1w) coronal rat brain MRI at days 5 (**left**) and 8 (**right**), overlaid with detection of BBB dysfunction voxels (BBBD, blue). (**C**) shows the results of the analysis of MRI T2w and T1w scans (reflecting edema and BBBD, respectively) conducted in animals exposed to five consecutive days of repeated low-frequency rTMS or treated with sham rTMS. No significant differences were found between the two groups on either day 5 (**left**) or 8 (**right**), confirming the safety of rTMS. (**D**) shows the results of repeated neurological assessments performed on days 9–12 (red line indicates the integer level of 18, indicating proper function). No difference was detected in NSS between rTMS-exposed and sham-treated rats (*n* = 7) at any of the time points. Data in (**C**) are expressed as median and IQR, and in D as means ± standard error of the mean.

We previously reported that rTMS-induced BBB opening in rats is transient and safe, and can facilitate the delivery of IGF-Trap into the brain [34]. Here, using a similar stimulation protocol [five consecutive days (days 1–5), one session per day], we aimed to further evaluate the safety and clinical potential of rTMS in a pre-clinical platform using both rat and mouse glioma models. To assess the safety of the procedure in a rat model, we applied the same stimulation protocol and conducted in vivo brain-MRI on days 5 and 8 following the last stimulation session (Figure 1A). Consistent with our previous findings, five days of low-frequency rTMS delivered to an intact scalp, either as real or sham stimulation, did not result in any measurable effects on both relative brain volumes of T2w hyper intensity [rTMS—4.475% (3.658–6.396), *n* = 13, and sham-treated—3.99% (2.961–6.216), *n* = 11, *p* = 0.89 as assessed by the Mann–Whitney test] and BBBD [rTMS—6.57% (3.135–7.857), *n* = 9 and sham-treated-5.09% (4.671–5.797), *n* = 8, *p* = 0.25, as assessed by the Mann–Whitney test] (Figure 1B and Figure 1C—left). Moreover, MRI performed on day 8 did not reveal a measurable difference between rats subjected to rTMS stimulations and the sham-treated group in either T2w hyper intensity [rTMS—5.37% (3.806–6.54), *n* = 10, and sham- 3.86% (3.053–4.81), *n* = 8, *p* = 0.17] or BBBD [rTMS—5.86% (4.547–7.071), *n* = 10, and sham—4.37% (3.196–7.485), *n* = 7, *p* = 0.25] (Figure 1B and Figure 1C—right). Neurological assessments performed on days 9–12 did not indicate any difference in NSS between rTMS (*n* = 7) and sham-treated (*n* = 7) rats at any of these time points (Figure 1D).

### 2.2. IGF-Trap Administration Coupled with rTMS Partially Inhibits C6-Glioma Tumor Progression

**Figure 2:**
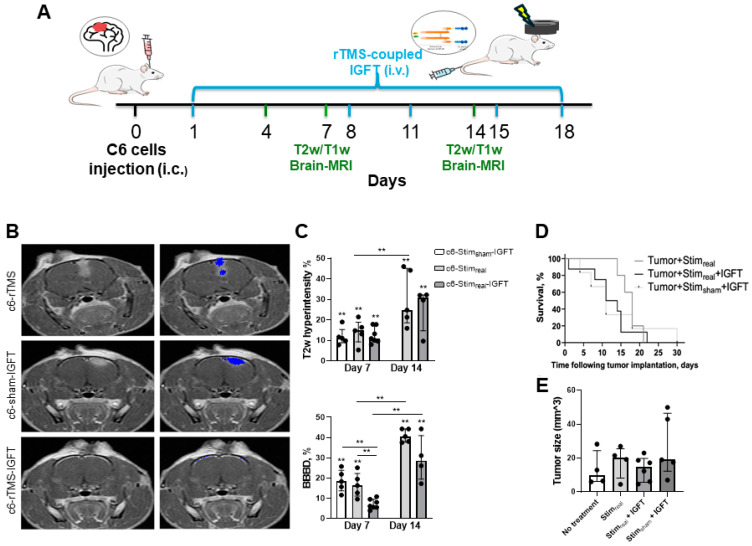
Intracerebral C6 tumor growth in rats is partially inhibited by the combination of rTMS and systemically administered IGF-Trap. (**A**) shows a diagrammatic representation of the experimental protocol. (**B**) shows representative contrast-enhanced T1-weighted coronal rat brain MRI images acquired 7 days following intracranial injection of C6 cells to animals subjected to rTMS alone (c6-rTMS, **top**), sham animals stimulated and injected intravenously with IGF-Trap (c6-sham—IGFT, **middle**), and animals subjected to rTMS and intravenously injected with IGF-Trap (c6-rTMS-IGFT, **bottom**). On the left are raw images and on the right are images overlaid with detection of voxels with BBB dysfunction. (**C**) shows relative volumes of T2w hyper-intensity (**top**) and BBBD (**bottom**) on days 7 and 14 post-C6 injection, calculated for c6-rTMS (dark gray), c6-sham-IGFT (light gray) and c6-rTMS-IGFT (black) rats compared to naïve animals. While c6-rTMS and c6-sham-IGFT rats exhibited increased relative BBBD volumes on day 7 compared to naïve animals, BBBD was significantly lower in the c6-rTMS-IGFT group. (**E**) shows median tumor sizes as evaluated by a radiologist based on T1w-MRI scans acquired on day 7. The difference in tumor size in the different treatment groups was not significant at that time point. (**D**) shows survival plots. Due to mortality, parameter extraction for c6-sham-IGFT was not feasible on day 14. Data in (**C**,**E**) are expressed as median and IQR, ** *p* < 0.01.

We previously confirmed that rTMS effectively induced transient opening of the BBB [33,34], resulting in increased delivery of anti-cancer drugs such as the IGF-Trap into the brain [12,34]. Therefore, after confirming the safety of rTMS, we tested the therapeutic efficacy of rTMS-coupled IGF-Trap administration in the C6-glioma tumor model. Three groups (rTMS, rTMS_sham_-IGFTrap and rTMS_real_-IGFTrap) were injected intra-cerebrally with C6 cells, followed by rTMS applied on days 1, 4, 8, 11, 15 and 18 post-tumor injection (see detailed description in Materials and Methods). Brain MRI scans, including T2w- and T1w-hyper-intensity sequences (Figure 2B), were conducted on days 7 and 14 post-implantation to evaluate tumor progression, and then compared to scans acquired from naïve animals that underwent repeated sham rTMS for five days (defined as naïve, *n* = 14). Scans acquired on day 7 revealed that c6-rTMS (*n* = 5), c6-rTMS-IGFT (*n* = 7) and c6-sham-IGFT (*n* = 5) animals had higher relative T2w-hyper-intense signal levels compared to naïve rats [14.75% (9.227–18.9), 10.9% (9.602–17.78) and 11.01% (8.8–15.31), *p* = 0.005, *p* = 0.001 and *p* = 0.004 respectively, as assessed by the Mann–Whitney test] (Figure 2C, top). However, while c6-rTMS and c6-sham-IGFT animals also exhibited increased T1w-hyper-intensity (i.e., BBBD) compared to naïve rats [16.37% (11.11–22.33) and 18.31% (13.66–23.84), *p* = 0.004 and *p* = 0.003, respectively, Mann–Whitney], BBBD values in the c6-rTMS-IGFT animals were similar to those in naïve rats [6.157% (5.537–9.512), *p* = 0.34, Mann–Whitney] and significantly lower than in c6-rTMS and c6-sham-IGFT rats (*p* = 0.007 and *p* = 0.004, respectively, Mann–Whitney) (Figure 2C, bottom), indicating partial efficacy of the combined treatment. On day 14, while c6-rTMS (*n* = 5) animals exhibited increased T2w-hyper-intensity compared to day 7 [24.77% (18.57–45.12), *p* = 0.047, Mann–Whitney), the T2w signal in c6-rTMS-IGFT rats (*n* = 4) did not significantly increase relative to day 7 [30.84% (14.65–32.14), day 14 vs. day 7—*p* = 0.089, Mann–Whitney] (Figure 2C, top), suggesting that rTMS-coupled IGF-Trap could slow tumor progression. Both c6-rTMS and c6-rTMS-IGFT exhibited increased BBBD on day 14 as compared to day 7 [40.57% (37.79–44.26), *p* = 0.009 and 28.62% (19.47–40.99), *p* = 0.008, respectively, Mann–Whitney) (Figure 2C, bottom). Tumor size calculated based on T1w-MRI scans acquired on day 7 also showed reduced tumor size in rats treated with rTMS and IGF-Trap (Figure 2E) relative to sham TMS treated rats, but the difference did not reach a statistically significant value. In addition, no significant differences in overall survival were found for any of the treatment groups (Figure 2D).

### 2.3. The rTMS Procedure Does Not Significantly Affect Intracerebral Tumor Growth in a Mouse Model of Glioma

**Figure 3:**
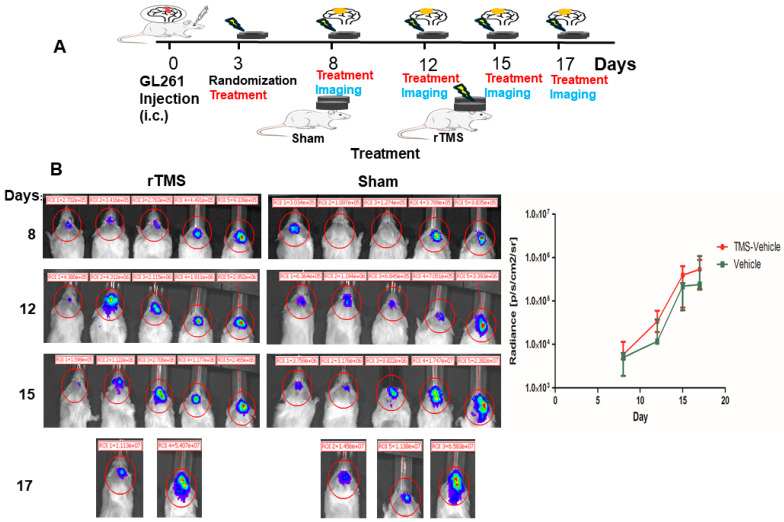
Lack of effect of rTMS on intracerebral tumor growth. Shown in (**A**) is a diagrammatic representation of the experimental protocol. Shown in ((**B**)—**left**) are optical images acquired following intracranial injection of 10^5^ GL261 cell, followed by bi-weekly rTMS administration from day 3 onward and (in (**B**)—**right**) the radiance per group (*n* = 5) expressed as median and IQR per treatment group.

Having observed increased IGF-Trap brain diffusion in a rat model, the TMS apparatus was subsequently adapted to administer rTMS to mice, in order to enable utilization of a mouse glioma model to assess the therapeutic benefit of administering IGF-Trap in conjunction with rTMS. The mouse-adapted rTMS setup can be seen in Supplementary Figure S1. We began by analyzing whether rTMS administration itself had an effect on intracranial tumor growth. Mice were injected with 10^5^ luciferase-expressing GL261 cells, randomized on day 3 post-tumor cell inoculation, with rTMS administered on day 3 and twice weekly thereafter until endpoint. Results shown in Figure 3 demonstrate that rTMS administration slightly accelerated tumor growth rate in the brain, but the effect was variable and did not significantly alter the outcome (survival).

### 2.4. rTMS When Combined with Systemic IGF-Trap Administration Partially Inhibited Tumor Growth

**Figure 4:**
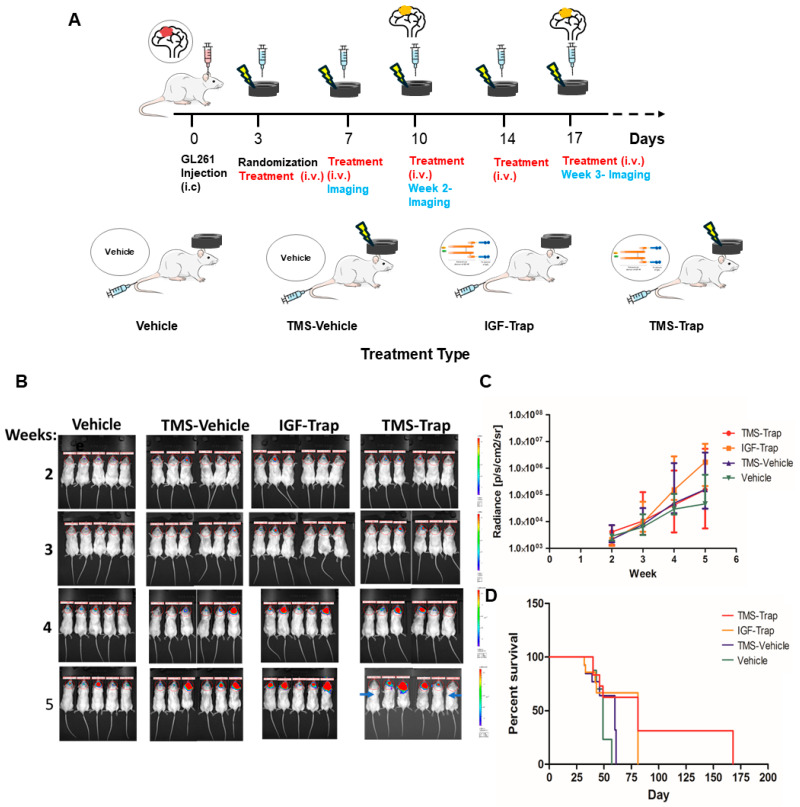
Systemic administration of IGF-Trap in conjunction with rTMS has a partial inhibitory effect on tumor growth and survival. GL261 (10^5^ cells/mouse) were injected orthotopically into NSG male mice (*n* = 5–6). The mice were randomized on day 3, at which time treatment began and continued twice weekly up to 57 days post tumor cell injection. The mice were injected intravenously with 10 mg/kg IGF-Trap, preceded (or not) by 5 rounds of TMS (1min pulses at 1Hz). Control mice received intravenous injections of vehicle (PBS) only. (**A**) shows a diagrammatic representation of the experimental protocol. (**B**) shows optical images of mice brains where the intensity of the signal, as represented in the color scales on the right, corresponds to tumor size and (**C**) shows the radiance expressed as median and IQR per group. (**D**) shows a Kaplan–Meier survival curve. Arrows in (**B**) denote mice that did not develop detectable tumors until 63 and 140 days post injection, and survived for 81 and 168 days, respectively.

We have previously shown that rTMS increased the diffusion of the IGF-Trap through the BBB [34]. To assess the therapeutic potential of IGF-Trap when administered systemically in conjunction with rTMS in a mouse glioma model, we injected mice intra-cranially with 10^5^ luciferase^+^ GL261 cells and randomized mice 3 days later for the following four treatment groups: (1) mice treated with vehicle only (PBS); (2) mice treated with vehicle immediately after TMS administration; (3) mice injected intravenously with 10 mg/kg IGF-Trap alone; and (4) mice injected with the same dose of IGF-Trap immediately following TMS administration. Treatment was administered twice weekly until day 57 post-tumor injection, when all mice in the vehicle-control group reached endpoint (morbidity). Intracerebral tumor growth was monitored once weekly using optical imaging (see diagrammatic representation in Figure 4A). We observed that, within 3 weeks post tumor cells inoculation, all mice in the vehicle-control- and IGF-Trap-injected groups had detectable tumors, whereas 2/6 mice treated with a combination of TMS and IGF-Trap had no detectable tumors at that time (Figure 4B,C). The majority of mice reached endpoint by 6–7 weeks post-tumor injection. However, despite termination of treatment on day 57, two mice in the combination therapy group had no detectable tumors for up to 63 and 140 days post tumor injection, respectively. A Kaplan–Meier survival curve is shown in Figure 4D. The median survival time increased from 49 days in the vehicle-injected group to 81 days in mice treated with a combination of TMS and IGF-Trap. However, the difference in overall survival in these mice, as compared to controls and assessed by the log-rank Mantel–Cox test, did not reach statistical significance (*p* = 0.122).

### 2.5. The Administration of IGF-Trap in Combination with rTMS Also Delays the Intracerebral Growth of a More Aggressive Variant of Glioma GL261

**Figure 5:**
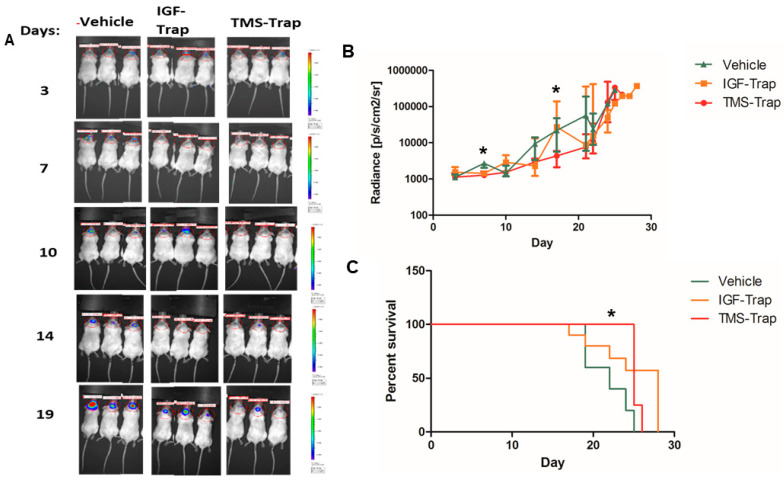
Tumor growth is delayed in mice treated with TMS prior to systemic IGF-Trap administration. Mice (*n* = 5) were injected intra-cerebrally with 3 × 10^4^ GL261v cells and randomized for treatment 3 days later. They received intravenous injections of 10 mg/kg IGF-Trap or vehicle (PBS) that were preceded (or not) with TMS administration 5 min earlier on day 3 and twice weekly thereafter until humane endpoint (morbidity). Tumor growth was monitored by optical imaging, performed once weekly following injection of luciferin. One mouse from the TMS/IGF-Trap treatment group was removed from the study due to a technical issue. For a diagrammatic depiction of the experimental protocol, see Figure 4. (**A**) shows representative mice from each treatment group, (**B**) shows the median and IQR for each group and (**C**) shows a Kaplan–Meier survival curve. * *p* < 0.05. as determined by the Mann–Whitney test (radiance) and the log-rank Mantel–Cox test (survival).

We performed a similar experiment using a variant of the GL261 cell line with a more rapid growth rate and aggressive phenotype, to shorten the treatment period. We found that, while the systemic administration of IGF-Trap alone did not alter tumor progression relative to vehicle-injected mice, the addition of rTMS prior to IGF-Trap injection delayed tumor growth in the treated mice for up to 22 days following glioma cell injection (Figure 5A). However, all mice eventually developed tumors that grew rapidly despite continued rTMS/IGF-Trap administration (Figure 5A,B). In this experiment, the median survival was 22, 28 and 25 days in vehicle-treated, IGF-Trap-treated and rTMS/IGF-Trap-treated mice, respectively. The overall survival, as compared to vehicle-treated mice and assessed by the log-rank Mantel–Cox test, significantly increased (*p* < 0.05) in the rTMS/IGF-Trap-treated animals but not in the IGF-Trap only-treated group (*p* = 0.0751).

## 3. Discussion

TMS administration is currently an FDA approved treatment for challenging psychiatric disorders, including major depressive disorder and treatment-resistant depression [29,31]. Multiple randomized controlled trials and the published literature have supported the safety and efficacy of rTMS as an antidepressant therapy [35] and its efficacy has been demonstrated in other neuropathological disorders such as neuropathic pain and stroke management [36]. A pre-clinical study has shown that rTMS may induce an increase in extracellular levels of the neurotransmitter glutamate [37], and in turn, the release of glutamate, upon TMS stimulation, which was found to transiently increase BBB permeability through the activation of NMDA receptors [33]. We have previously shown that rTMS applied at high intensity increased the diffusion of the IGF-signaling inhibitor, the IGF-Trap through the BBB [34]. However, the benefit of rTMS in the treatment of brain malignancies remained to be demonstrated. Here, we report that systemic IGF-Trap administration, when combined with rTMS, had beneficial therapeutic effects on intra-cerebral tumor growth in two different cancer models used in two different animal species. However, the response to this combinatorial protocol was variable and, while tumor appearance was delayed in some mice, the effect was generally transient and tumors in both rats and mice eventually became resistant to the treatment and progressed to endpoint. This may be due to several factors and limitations, including a limited impact of rTMS on the BBB, potentially not allowing sufficient levels of IGF-Trap to reach the brain and intra-tumor heterogeneity of glioblastoma cells—a characteristic of these tumors [2] with pre-existing subpopulations within the tumors that were not sensitive to IGF-signaling inhibition. Continuous treatment with the IGF-Trap may have selected cells with upregulated compensatory survival and growth mechanisms that rescued the cells from IGF-signaling blockade, as we have previously found for triple negative breast cancer cells [38]. Alternatively, it is possible that efflux transporters or other clearance mechanisms reduced the effective concentration of IGF-Trap that reached the tumor. It is also possible that the effect of rTMS on the BBB may have been partial, thus not allowing sufficient IGF-Trap levels to gain access to the brain and eliminate all tumor cells. The possibility that endothelial cells associated with the BBB eventually developed some resistance to repetitive TMS application can also not be ruled out. Finally, it should be noted that tumor progression in both rats and mice was measured indirectly via radiological and optical imaging, respectively, and these methods may not be sensitive enough to detect subtle differences in the progression of individual tumors. Notably, however, in both rats and mice, rTMS was safe; no deleterious effects were observed in the treated animals, consistent with the safety profile of this treatment in humans [35].

A pilot study of 15 patients with malignant glial tumors showed increased BBB permeability following TMS, especially around the tumor bed [33]. However, cancerous cells are known to release glutamate that can cause excite-toxic death to surrounding brain cells, in order to expand in the brain [39]. It was also documented that the BBB is disrupted in high-grade tumors; this is often a sign of other physiological changes, such as angiogenesis [40]. This may explain why, in some mice treated only with systemically administered IGF-Trap, we also observed a decrease in tumor growth and improved survival. In addition, IGF-Trap may reduce the systemic levels of IGF-1 and IGF-2, thereby reducing the pool of IGF in the brain.

The C6 cells are chemically induced rat brain cancer cells, which are widely used as a glioma model for in vitro and in vivo studies [41]. In recent years, the relevance of these cells as a model for human glioma has been questioned because their origin as chemically induced brain tumors may better represent “gliosarcomas” [42]. It should be noted, however, that in different studies, both the phenotype and immune microenvironment of experimental rat C6 gliomas were shown to resemble those of human glioblastomas [43,44]. It is also worth noting that the clinical features and outcomes of gliosarcoma and glioblastoma, as revealed in clinical studies, are essentially indistinguishable [45]. Previously, using a genetic approach, we have already shown that the growth of these cells in the brain can be inhibited by blocking IGF-1R expression [13]. We show here that these cells also respond when targeted intra-cerebrally with an IGF-1R inhibitor, although the response was partial. In patients treated with TMS, a variable response was also observed, as approximately 40% of patients did not respond to maintenance treatment [46]. Future optimization of the TMS technology may be required to improve the efficacy of this technology for the delivery of targeted drugs to the brain. For example, changes in parameters such as stimulation frequency, stimulation amplitude, train duration, inter-train intervals, and coil characteristics may impact the extent and characteristics of the BBB opening.

In this study, we used the IGF-Trap as a stand-alone therapy. Our results show that glioma cells respond to this agent if it is effectively delivered to the brain. In the clinical setting, however, a biologic drug such as the IGF-Trap is more likely to be used in combination with other modalities, such as chemotherapy and radiation, in first-, and more likely, second-line regimens, where it is expected to improve the efficacy of the standard of care treatments. In this scenario, the effect of IGF-Trap and rTMS may be optimized and more sustained, improving the outcome. Moreover, the clinical use of rTMS in neuro-oncology to improve the quality of life for cancer patients is an area of growing interest, and all studies to date have indicated a favorable safety profile. A recent review into the use of rTMS for neuro-rehabilitation in patients with brain tumors has shown positive outcomes [47], with no incidence of seizures. Similarly, the use of navigated rTMS in a randomized clinical trial has been shown to improve outcomes in post-surgical paresis in glioma patients [48]. Although caution is essential when applying brain stimulation to patients, the results to date indicate that, if used appropriately, rTMS could develop into a beneficial application in the neuro-oncological space.

Furthermore, the ability of rTMS to improve the delivery and therapeutic effect of a large biologic, such as the IGF-Trap, without deleterious effects, bodes well for the use of this method to deliver other biologics, such as antibodies, for the treatment of glioma, as well as the management of brain metastases that originated from other primary tumors.

## 4. Materials and Methods

### 4.1. Cells

The rat C6 cells were derived from a chemically induced rat brain cancer and are widely used as a rat glioma model for in vitro and in vivo studies [13,41]. The cells were obtained from the American Type Culture Collection (ATCC) and were cultured and maintained in the laboratory of Prof. Varda Shoshan-Barmatz at the National Institute for Biotechology of the Negev (Ben-Gurion University of the Negev).

The murine GL261 glioma cell line was obtained from the DTP/DCTD/NCI Tumor Repository and used in all mouse experiments unless otherwise indicated. The use of these cells as a GBM model has been extensively reviewed elsewhere [49]. A more aggressive variant of these cells was originally obtained from Dr. X.O. Breakefield (Harvard Medical School, Boston, MA, USA) [50] and provided to our laboratory courtesy of Dr. Janusz Rak (Research Institute—McGill University Health Center) [51]. The cells were cultured at 37 °C in DMEM medium (Wisent, Saint-Jean-Baptiste, QC, Canada) supplemented with 10% heat-inactivated fetal bovine serum FBS (Wisent) and antibiotics. As per the McGill University Animal Care Committee and the McGill University Biohazard Committee guidelines, the cells were periodically tested for mouse and human pathogens and mycoplasma infection, and were last retested in 2021–2022 during the course of these studies. To avoid cross-contamination and phenotype changes, these cells were not propagated in long-term cultures. Following acquisition from the tumor repositories, they were expanded, maintained as frozen stocks and cultured for 2 to 4 weeks only prior to their injection to preserve the tumorigenic phenotype. To prepare the cells for injection, they were cultured overnight in a full medium and then washed twice in a serum-free medium before the injection, as described below.

### 4.2. Animals

Rats: Male Sprague–Dawley rats were obtained from Harlan Laboratories Israel Ltd. (Jerusalem, Israel) and maintained in the animal facility of the Ben-Gurion University (Beer Sheva, Israel). All experimental procedures in rats (body weight 232.73 ± 42.22 gr) were approved by the Ben-Gurion University ethics committee for animal experiments (Protocol no. 04-01-2020), according to the guidelines of the Israeli Council for Animal Experimentation. Unless otherwise specified, all materials were purchased from Sigma-Aldrich Ltd. (Rehovot, Israel). The rats were randomly divided into the indicated experimental groups according to treatment protocols. Data were analyzed identically for all treatment groups in a blind fashion.

Mice: All mouse experiments were carried out in strict accordance with the guidelines of the Canadian Council on Animal Care (CCAC) ‘‘Guide to the Care and Use of Experimental Animals’’ and under the conditions and procedures approved by the Animal Care Committee of McGill University (AUP number: 5733). All mice were bred in the animal facility of the Research Institute of the McGill University Health Center and used for experiments at the ages of 7–12 weeks old. The experiments were performed in male NSG mice that were orthotopically injected with the murine glioma GL261 cells.

### 4.3. The IGF-Trap

The IGF-Trap is a fusion protein consisting of the entire extracellular domain of the human IGF-1R fused to the Fc portion of human IgG_1_ (See diagrammatic representation in Figure 2A). Similarly to the cognate receptor, the IGF-Trap binds the ligands IGF-1 and IGF-2 (but not insulin) with high affinity, reducing ligand bioavailability in the circulation and the tumor microenvironment. The bioengineering and properties of the IGF-Trap were described in detail previously [15], and were also reviewed extensively in [14]. The bioengineering of a 3rd generation IGF-Trap with improved physicochemical and pharmacodynamic properties was described recently [16]. This Trap (IGF-Trap 3.3) was used in all of the experiments described herein.

### 4.4. Intracerebral Tumor Cell Injections

Rat protocol: Prior to intracerebral injection, the rats were anesthetized by intraperitoneal administration of ketamine (100 mg/mL, 75 mg/kg) and xylazine (20 mg/mL, 5mg/kg) followed by isoflurane (1–2% in 100% O_2_) inhalation. A sagittal incision was made, and a cranial hole was drilled at 1mm anterior, 1.5mm right to Bregma. A beveled micro-syringe (World Precision Instruments Ltd., Sarasota, FL, USA) containing 10^6^ C6 cells in 10 µL was advanced through the hole to a depth of 3.9mm from the skull surface and then withdrawn by 0.5mm. The cell suspension was delivered at a rate of 1 µL/min. On days 7 and 14 following tumor implantation, brain MRI (Aspect Ltd., Shoham, Israel) scans were conducted under anesthesia (1–2% isoflurane in 100% O_2_).

Mouse protocol: rTMS was administered 5 min prior to intravenous injection of the IGF-Trap at the doses indicated. The mice were first anesthetized using a flowmeter (0.4 to 0.8 L/min) and isoflurane vaporizer (2 to 2.5%). The mice were placed in a holder specifically adapted for mouse rTMS administration and the bespoke TMS coil (modified butterfly TMS coil with a customizable tilt angle between wings; elliptical coil, long-axis 6.2 cm, short-axis 3.3cm; coil thickness 0.8cm, 12 turns of copper wire encased in epoxy, designed by BrainsWay Ltd., Jerusalem, Israel) with a coil inductance of 18 µH (see image and video in Supplementary Figure S1). The coil was connected to a Rapid2 stimulator (Magstim Ltd., Oxford, UK). After establishing the motor response threshold, the mice received 1Hz rTMS administered at 130% of the resting motor threshold (rMT) [33]. It is important to note that this represents an intensity significantly higher than commonly used for clinical treatment protocols. Repetitive stimulation consisting of 50 s train duration, 60 s inter-train interval, 5 trains and 250 pulses in total were administered per mouse. Sham rTMS was administered by diverting stimulator current from the head coil to an adjacent coil, placed 12 cm away, that did not elicit any motor response.

### 4.5. MRI

MRI was used to monitor intracerebral tumor development in rats using anatomical and permeability imaging. Anatomical state was evaluated using fast spin-echo T2-weighted (T2w) imaging (TR/TE/NEX  =  3400 ms/74 ms/4) [52,53] and spin-echo T1-weighted scans (TR/TE/NEX  =  400 ms/14 ms/2). Image segmentation was applied offline using an in-house developed MATLAB [34], calculating brain and peripheral signal histograms in T2w imaging and identifying voxel intensity within brain signal range and out of peripheral signal range. Offline image analysis was carried out, following segmentation, using in-house developed MATLAB algorithms [34].

For detection of a lesion (as edema), the volume of the T2w hyper-intense brain signal was measured. The signal histogram in a 3 × 3 environment around each voxel (environmental signal) was calculated and compared to the signal histogram of a reference control region acquired from the hemisphere contralateral to the stimulated/tumor-implanted hemisphere [34]. Voxels with a hyper-intense signal were defined as those having a higher self and mean environmental signal than the mean control signal (according to Mann–Whitney U, MWU rank). The relative T2w hyper-intense signal level was calculated as the number of hyper-intense voxels relative to the total number of brain voxels.

BBB permeability was evaluated using a contrast-enhanced scan protocol (CE-MRI) [34,54]. Two T1-weighted scans were performed, in which the gadolinium (Gd)-containing contrast agent, gadoteric acid (DOTAREM, Guerbet Ltd., Villepinte, France), was administered i.m. (1 mL/kg), following the first scan and 30 min prior to the second scan. Offline image registration [55] and segmentation were performed. The environmental signal histogram, using contrast-enhanced imaging, was calculated and compared to the signal histogram of a reference region acquired from the temporal muscle [34], and the parallel environmental signal histogram acquired from the first, non-contrast-enhanced imaging session. BBB dysfunction (BBBD) voxels were identified as having a higher (according to MWU rank) self and mean environmental contrast-enhanced signal than the non-contrast-enhanced parallel signal, and a higher mean environmental contrast-enhanced signal than the mean reference signal. The relative BBBD volume (# of BBBD voxels/# of brain voxels) was calculated.

### 4.6. Tumor Size Measurement

Tumor size in rats was measured by an experienced radiologist. Measurements, using a volumetric tool (Carestream Veu 12.1 Carestream health, Rochester, NY, USA), were performed based on T1w MRI scans conducted 7 days following intracerebral C6 glioma cell implantation.

### 4.7. Neurological Severity Score (NSS)

Neurological evaluations were conducted on days 9–12 post rTMS using the neurological severity scoring scale (NSS) [56]. Briefly, basic motor and sensory skills such as walking, balance and reflex were visually evaluated. The specific parameters evaluated were the following: (1) flexion of forelimb/hindlimb and head tilt by >10° when animals are raised by their tail; (2) walking; (3) sensory test—pinching of hind limb to evoke stimulus; (4) reflex in response to auditory stimulus, i.e., pinna/corneal/startle; (5) seizures in response to auditory stimulus; and (6) balance upon placement on wire mesh. Parameters were scored based on an integer scale of 0–18, with 0 indicating poor and 18 indicating proper function.

### 4.8. Statistical Analysis

Unless otherwise indicated, data are expressed as median and interquartile range (IQR). All statistical analyses were performed using GraphPad Prism Software, LLC. Survival was assessed using the Kaplan–Meier estimator [57] and the chi-squared and the log rank Mantel–Cox tests were used for analysis, as indicated. The Mann–Whitney U test was used for all other analyses. *p* ≤ 0.05 was defined as statistical significance.

## 5. Conclusions

1. Repetitive TMS could increase the anti-tumor effect of systemically administered IGF-Trap in rat and mouse orthotopic glioma models.
2. The tumor growth inhibitory effect of IGF-Trap administered in combination with rTMS was transient and most evident in the early stages following intracerebral injection of glioma cells.
3. Further optimization of the combinatorial treatment and the addition of chemotherapy and radiation may improve treatment outcome.

## Data Availability

The authors declare that the data supporting the findings of this study are available within the paper. Unprocessed (raw) data can be made available by the authors upon reasonable request.

## Supplementary Materials

The following supporting information can be downloaded at: https://www.mdpi.com/article/10.3390/ph17121607/s1, Figure S1: Adaptation of the TMS technology for use in mice. Shown is the dTMS apparatus adapted by Brainsway for extra-cranial stimulation of mice (**A**). TMS may be applied as a single pulse, or repeatedly (rTMS), with variation in intensity, site and orientation of the magnetic field. Deep TMS (dTMS) is a novel form of TMS that uses specialized coils (seen in photograph, A&B) designed to reach broader and deeper areas within the brain.

## References

[1] Alexander B.M., Cloughesy T.F. (2017). Adult Glioblastoma. J. Clin. Oncol..

[2] Wang L.B., Karpova A., Gritsenko M.A., Kyle J.E., Cao S., Li Y., Rykunov D., Colaprico A., Rothstein J.H., Hong R. (2021). Proteogenomic and metabolomic characterization of human glioblastoma. Cancer Cell.

[3] Crespo I., Vital A.L., Gonzalez-Tablas M., Patino Mdel C., Otero A., Lopes M.C., de Oliveira C., Domingues P., Orfao A., Tabernero M.D. (2015). Molecular and Genomic Alterations in Glioblastoma Multiforme. Am. J. Pathol..

[4] Pearson J.R.D., Regad T. (2017). Targeting cellular pathways in glioblastoma multiforme. Signal Transduct. Target. Ther..

[5] Maris C., D’Haene N., Trépant A.L., Le Mercier M., Sauvage S., Allard J., Rorive S., Demetter P., Decaestecker C., Salmon I. (2015). IGF-IR: A new prognostic biomarker for human glioblastoma. Br. J. Cancer.

[6] Tirro E., Massimino M., Romano C., Martorana F., Pennisi M.S., Stella S., Pavone G., Di Gregorio S., Puma A., Tomarchio C. (2020). Prognostic and Therapeutic Roles of the Insulin Growth Factor System in Glioblastoma. Front. Oncol..

[7] Ma Y., Tang N., Thompson R.C., Mobley B.C., Clark S.W., Sarkaria J.N., Wang J. (2016). InsR/IGF1R Pathway Mediates Resistance to EGFR Inhibitors in Glioblastoma. Clin. Cancer Res..

[8] Song K., Yuan Y., Lin Y., Wang Y.X., Zhou J., Gai Q.J., Zhang L., Mao M., Yao X.X., Qin Y. (2018). ERBB3, IGF1R, and TGFBR2 expression correlate with PDGFR expression in glioblastoma and participate in PDGFR inhibitor resistance of glioblastoma cells. Am. J. Cancer Res..

[9] Samani A.A., Yakar S., LeRoith D., Brodt P. (2007). The role of the IGF system in cancer growth and metastasis: Overview and recent insights. Endocr. Rev..

[10] Seccareccia E., Brodt P. (2012). The role of the insulin-like growth factor-I receptor in malignancy: An update. Growth Horm. IGF Res..

[11] Baserga R. (2005). The insulin-like growth factor-I receptor as a target for cancer therapy. Expert. Opin. Ther. Targets.

[12] Chen Y.M., Leibovitch M., Zeinieh M., Jabado N., Brodt P. (2023). Targeting the IGF-Axis in Cultured Pediatric High-Grade Glioma Cells Inhibits Cell Cycle Progression and Survival. Pharmaceuticals.

[13] Samani A.A., Nalbantoglu J., Brodt P. (2020). Glioma Cells with Genetically Engineered IGF-I Receptor Downregulation Can Persist in the Brain in a Dormant State. Front. Oncol..

[14] Chen Y.M., Qi S., Perrino S., Hashimoto M., Brodt P. (2020). Targeting the IGF-Axis for Cancer Therapy: Development and Validation of an IGF-Trap as a Potential Drug. Cells.

[15] Wang N., Rayes R.F., Elahi S.M., Lu Y., Hancock M.A., Massie B., Rowe G.E., Aomari H., Hossain S., Durocher Y. (2015). The IGF-Trap: Novel inhibitor of carcinoma growth and metastasis. Mol. Cancer Ther..

[16] Vaniotis G., Moffett S., Sulea T., Wang N., Elahi S.M., Lessard E., Baardsnes J., Perrino S., Durocher Y., Frystyk J. (2018). Enhanced anti-metastatic bioactivity of an IGF-TRAP re-engineered to improve physicochemical properties. Sci. Rep..

[17] Dong X. (2018). Current Strategies for Brain Drug Delivery. Theranostics.

[18] Pardridge W.M. (2012). Drug transport across the blood-brain barrier. J. Cereb. Blood Flow Metab..

[19] Rapoport S.I. (2000). Osmotic opening of the blood-brain barrier: Principles, mechanism, and therapeutic applications. Cell Mol. Neurobiol..

[20] van Vliet E.A., da Costa Araujo S., Redeker S., van Schaik R., Aronica E., Gorter J.A. (2007). Blood-brain barrier leakage may lead to progression of temporal lobe epilepsy. Brain.

[21] Hynynen K., McDannold N., Vykhodtseva N., Raymond S., Weissleder R., Jolesz F.A., Sheikov N. (2006). Focal disruption of the blood-brain barrier due to 260-kHz ultrasound bursts: A method for molecular imaging and targeted drug delivery. J. Neurosurg..

[22] Kinoshita M., McDannold N., Jolesz F.A., Hynynen K. (2006). Noninvasive localized delivery of Herceptin to the mouse brain by MRI-guided focused ultrasound-induced blood-brain barrier disruption. Proc. Natl. Acad. Sci. USA.

[23] McDannold N., Vykhodtseva N., Hynynen K. (2006). Targeted disruption of the blood-brain barrier with focused ultrasound: Association with cavitation activity. Phys. Med. Biol..

[24] Abrahao A., Meng Y., Llinas M., Huang Y., Hamani C., Mainprize T., Aubert I., Heyn C., Black S.E., Hynynen K. (2019). First-in-human trial of blood-brain barrier opening in amyotrophic lateral sclerosis using MR-guided focused ultrasound. Nat. Commun..

[25] Rezai A.R., Ranjan M., D’Haese P.F., Haut M.W., Carpenter J., Najib U., Mehta R.I., Chazen J.L., Zibly Z., Yates J.R. (2020). Noninvasive hippocampal blood-brain barrier opening in Alzheimer’s disease with focused ultrasound. Proc. Natl. Acad. Sci. USA.

[26] Cho E.E., Drazic J., Ganguly M., Stefanovic B., Hynynen K. (2011). Two-photon fluorescence microscopy study of cerebrovascular dynamics in ultrasound-induced blood-brain barrier opening. J. Cereb. Blood Flow Metab..

[27] Kovacs Z.I., Kim S., Jikaria N., Qureshi F., Milo B., Lewis B.K., Bresler M., Burks S.R., Frank J.A. (2017). Disrupting the blood-brain barrier by focused ultrasound induces sterile inflammation. Proc. Natl. Acad. Sci. USA.

[28] Zangen A., Roth Y., Voller B., Hallett M. (2005). Transcranial magnetic stimulation of deep brain regions: Evidence for efficacy of the H-coil. Clin. Neurophysiol..

[29] Levkovitz Y., Isserles M., Padberg F., Lisanby S.H., Bystritsky A., Xia G., Tendler A., Daskalakis Z.J., Winston J.L., Dannon P. (2015). Efficacy and safety of deep transcranial magnetic stimulation for major depression: A prospective multicenter randomized controlled trial. World Psychiatry.

[30] Pell G.S., Harmelech T., Zibman S., Roth Y., Tendler A., Zangen A. (2022). Efficacy of Deep TMS with the H1 Coil for Anxious Depression. J. Clin. Med..

[31] Carmi L., Tendler A., Bystritsky A., Hollander E., Blumberger D.M., Daskalakis J., Ward H., Lapidus K., Goodman W., Casuto L. (2019). Efficacy and Safety of Deep Transcranial Magnetic Stimulation for Obsessive-Compulsive Disorder: A Prospective Multicenter Randomized Double-Blind Placebo-Controlled Trial. Am. J. Psychiatry.

[32] Zangen A., Moshe H., Martinez D., Barnea-Ygael N., Vapnik T., Bystritsky A., Duffy W., Toder D., Casuto L., Grosz M.L. (2021). Repetitive transcranial magnetic stimulation for smoking cessation: A pivotal multicenter double-blind randomized controlled trial. World Psychiatry.

[33] Vazana U., Veksler R., Pell G.S., Prager O., Fassler M., Chassidim Y., Roth Y., Shahar H., Zangen A., Raccah R. (2016). Glutamate-Mediated Blood-Brain Barrier Opening: Implications for Neuroprotection and Drug Delivery. J. Neurosci..

[34] Vazana U., Schori L., Monsonego U., Swissa E., Pell G.S., Roth Y., Brodt P., Friedman A., Prager O. (2020). TMS-Induced Controlled BBB Opening: Preclinical Characterization and Implications for Treatment of Brain Cancer. Pharmaceutics.

[35] McClintock S.M., Reti I.M., Carpenter L.L., McDonald W.M., Dubin M., Taylor S.F., Cook I.A., O’Reardon J., Husain M.M., Wall C. (2018). Consensus Recommendations for the Clinical Application of Repetitive Transcranial Magnetic Stimulation (rTMS) in the Treatment of Depression. J. Clin. Psychiatry.

[36] Lefaucheur J.P., Aleman A., Baeken C., Benninger D.H., Brunelin J., Di Lazzaro V., Filipovic S.R., Grefkes C., Hasan A., Hummel F.C. (2020). Evidence-based guidelines on the therapeutic use of repetitive transcranial magnetic stimulation (rTMS): An update (2014–2018). Clin. Neurophysiol..

[37] Zangen A., Hyodo K. (2002). Transcranial magnetic stimulation induces increases in extracellular levels of dopamine and glutamate in the nucleus accumbens. Neuroreport.

[38] Tsui J., Qi S., Perrino S., Leibovitch M., Brodt P. (2021). Identification of a Resistance Mechanism to IGF-IR Targeting in Human Triple Negative MDA-MB-231 Breast Cancer Cells. Biomolecules.

[39] Sontheimer H. (2008). A role for glutamate in growth and invasion of primary brain tumors. J. Neurochem..

[40] Provenzale J.M., Mukundan S., Dewhirst M. (2005). The role of blood-brain barrier permeability in brain tumor imaging and therapeutics. AJR Am. J. Roentgenol..

[41] Grobben B., De Deyn P.P., Slegers H. (2002). Rat C6 glioma as experimental model system for the study of glioblastoma growth and invasion. Cell Tissue Res..

[42] Huszthy P.C., Daphu I., Niclou S.P., Stieber D., Nigro J.M., Sakariassen P.O., Miletic H., Thorsen F., Bjerkvig R. (2012). In vivo models of primary brain tumors: Pitfalls and perspectives. Neuro Oncol..

[43] Gieryng A., Pszczolkowska D., Bocian K., Dabrowski M., Rajan W.D., Kloss M., Mieczkowski J., Kaminska B. (2017). Immune microenvironment of experimental rat C6 gliomas resembles human glioblastomas. Sci. Rep..

[44] Jacobs V.L., Valdes P.A., Hickey W.F., De Leo J.A. (2011). Current review of in vivo GBM rodent models: Emphasis on the CNS-1 tumour model. ASN Neuro.

[45] Zhang G., Huang S., Zhang J., Wu Z., Lin S., Wang Y. (2016). Clinical outcome of gliosarcoma compared with glioblastoma multiforme: A clinical study in Chinese patients. J. Neuro-Oncol..

[46] Connolly K.R., Helmer A., Cristancho M.A., Cristancho P., O’Reardon J.P. (2012). Effectiveness of transcranial magnetic stimulation in clinical practice post-FDA approval in the United States: Results observed with the first 100 consecutive cases of depression at an academic medical center. J. Clin. Psychiatry.

[47] Einstein E.H., Dadario N.B., Khilji H., Silverstein J.W., Sughrue M.E., D’Amico R.S. (2022). Transcranial magnetic stimulation for post-operative neurorehabilitation in neuro-oncology: A review of the literature and future directions. J. Neuro-Oncol..

[48] Ille S., Kelm A., Schroeder A., Albers L.E., Negwer C., Butenschoen V.M., Sollmann N., Picht T., Vajkoczy P., Meyer B. (2021). Navigated repetitive transcranial magnetic stimulation improves the outcome of postsurgical paresis in glioma patients—A randomized, double-blinded trial. Brain Stimul..

[49] Haddad A.F., Young J.S., Amara D., Berger M.S., Raleigh D.R., Aghi M.K., Butowski N.A. (2021). Mouse models of glioblastoma for the evaluation of novel therapeutic strategies. Neuro-Oncol. Adv..

[50] van Solinge T.S., Friis K.P., O’Brien K., Verschoor R.L., van Aarle J., Koekman A., Breakefield X.O., Vader P., Schiffelers R., Broekman M. (2023). Heparin interferes with the uptake of liposomes in glioma. Int. J. Pharm. X.

[51] Daniel P., Meehan B., Sabri S., Jamali F., Sarkaria J.N., Choi D., Garnier D., Kitange G., Glennon K.I., Paccard A. (2022). Detection of temozolomide-induced hypermutation and response to PD-1 checkpoint inhibitor in recurrent glioblastoma. Neuro-Oncol. Adv..

[52] Bar-Klein G., Lublinsky S., Kamintsky L., Noyman I., Veksler R., Dalipaj H., Senatorov V.V., Swissa E., Rosenbach D., Elazary N. (2017). Imaging blood-brain barrier dysfunction as a biomarker for epileptogenesis. Brain.

[53] Lippmann K., Kamintsky L., Kim S.Y., Lublinsky S., Prager O., Nichtweiss J.F., Salar S., Kaufer D., Heinemann U., Friedman A. (2017). Epileptiform activity and spreading depolarization in the blood-brain barrier-disrupted peri-infarct hippocampus are associated with impaired GABAergic inhibition and synaptic plasticity. J. Cereb. Blood Flow Metab..

[54] Chassidim Y., Veksler R., Lublinsky S., Pell G.S., Friedman A., Shelef I. (2013). Quantitative imaging assessment of blood-brain barrier permeability in humans. Fluids Barriers CNS.

[55] Guizar-Sicairos M., Thurman S.T., Fienup J.R. (2008). Efficient subpixel image registration algorithms. Opt. Lett..

[56] Teichberg V.I., Cohen-Kashi-Malina K., Cooper I., Zlotnik A. (2009). Homeostasis of glutamate in brain fluids: An accelerated brain-to-blood efflux of excess glutamate is produced by blood glutamate scavenging and offers protection from neuropathologies. Neuroscience.

[57] Goel M.K., Khanna P., Kishore J. (2010). Understanding survival analysis: Kaplan-Meier estimate. Int. J. Ayurveda Res..

