# Supplementary material for: Transcranial Magnetic Stimulation Enhances the Therapeutic Effect of IGF-Trap in Intracerebral Glioma Models
Source: Pharmaceuticals (Basel). 2024 Nov 28;17(12):1607. doi: 10.3390/ph17121607 (PMC11677529; doi:10.3390/ph17121607)
Supplement: Supplementary file 1 [file pharmaceuticals-17-01607-s001.zip › pharmaceuticals-3194640-supplementary.pptx]

## Slide 1
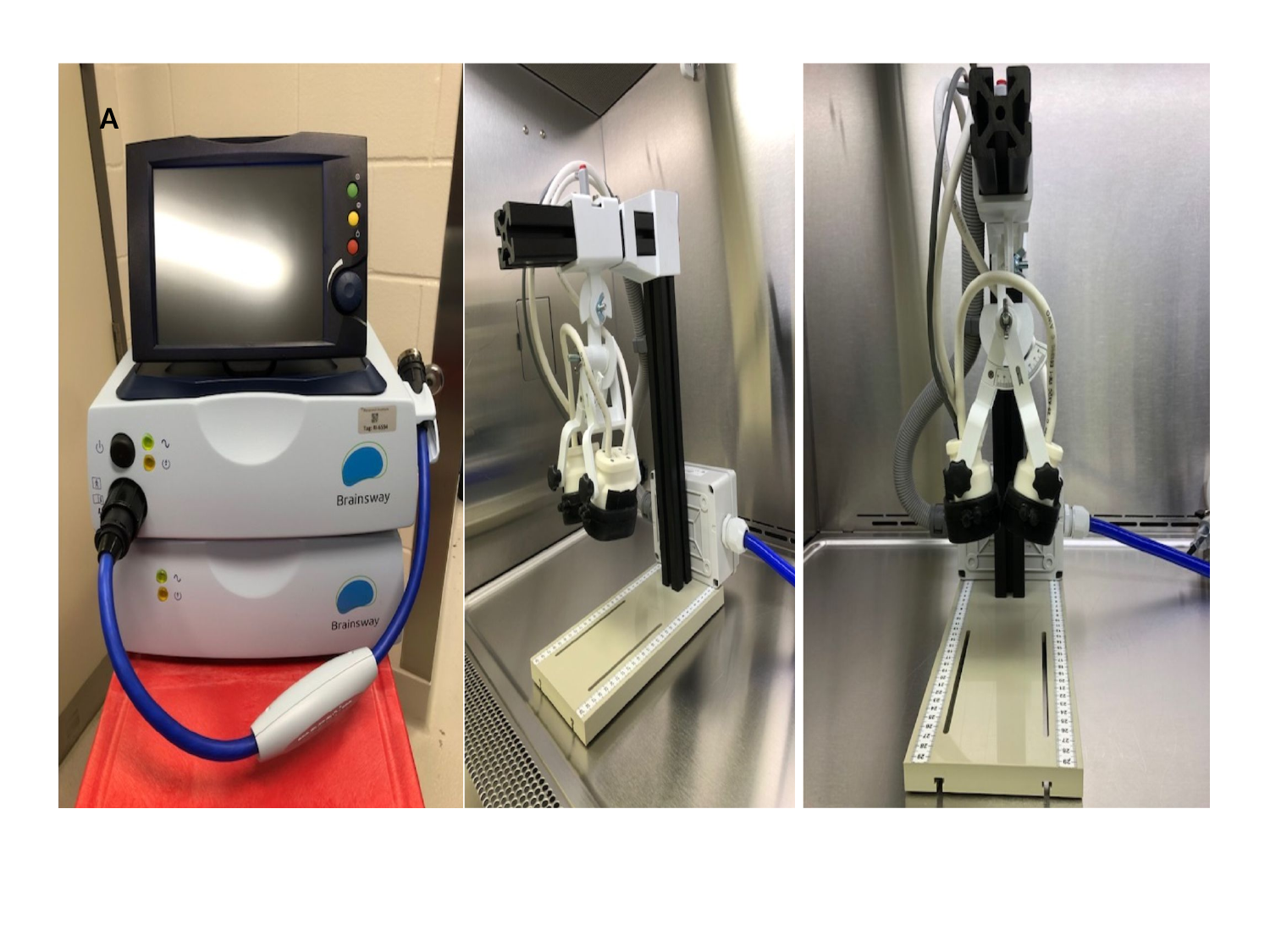

A

## Slide 2
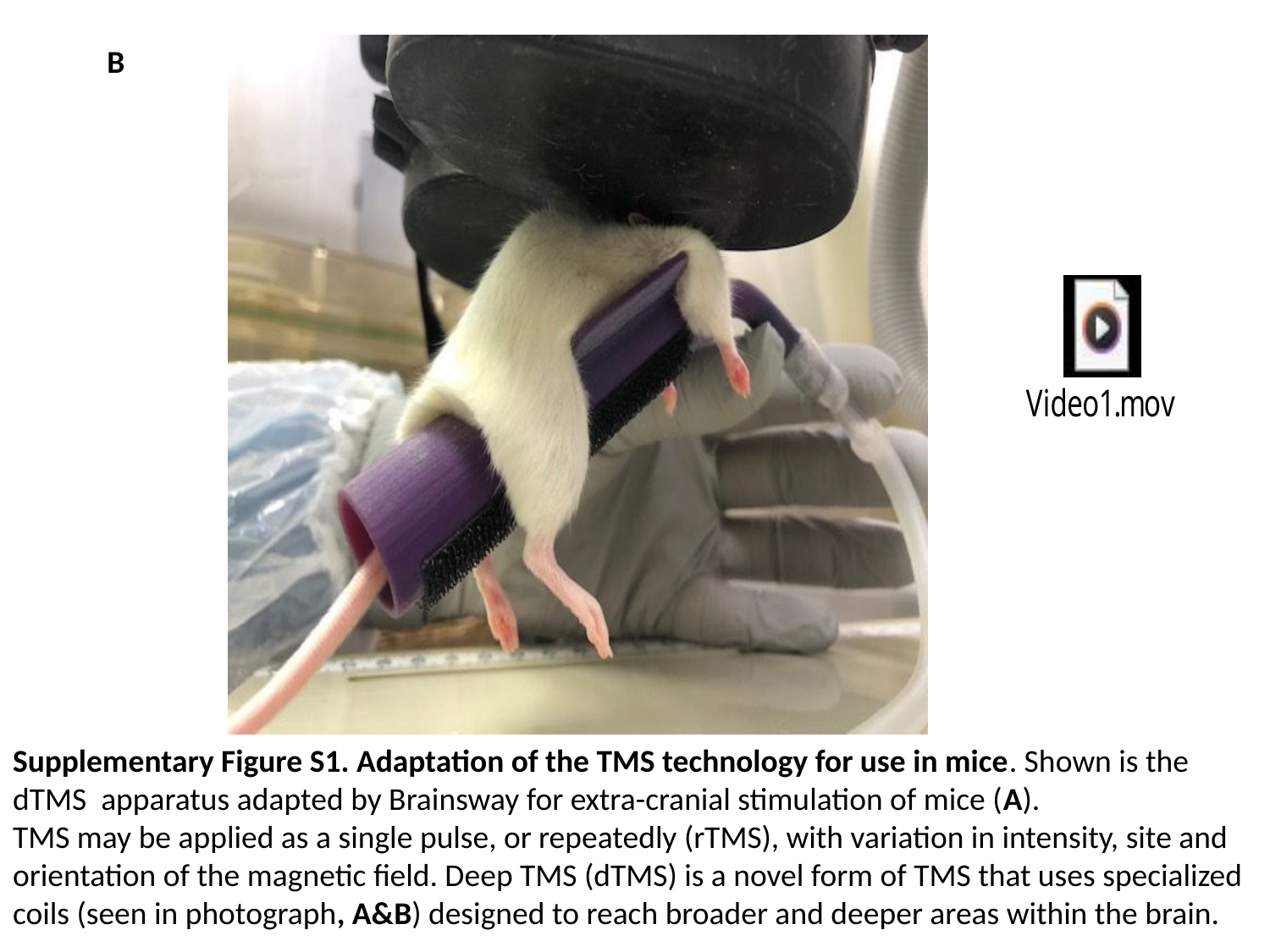

B
Supplementary Figure S1. Adaptation of the TMS technology for use in mice. Shown is the dTMS apparatus adapted by Brainsway for extra-cranial stimulation of mice (A).
TMS may be applied as a single pulse, or repeatedly (rTMS), with variation in intensity, site and orientation of the magnetic field. Deep TMS (dTMS) is a novel form of TMS that uses specialized coils (seen in photograph, A&B) designed to reach broader and deeper areas within the brain.
